# The PIK3CA H1047R Mutation Confers Resistance to BRAF and MEK Inhibitors in A375 Melanoma Cells through the Cross-Activation of MAPK and PI3K–Akt Pathways

**DOI:** 10.3390/pharmaceutics14030590

**Published:** 2022-03-08

**Authors:** Saverio Candido, Rossella Salemi, Sara Piccinin, Luca Falzone, Massimo Libra

**Affiliations:** 1 Department of Biomedical and Biotechnological Sciences, University of Catania, 95123 Catania, Italy; scandido@unict.it (S.C.); rossellasalemi2580@gmail.com (R.S.); m.libra@unict.it (M.L.); 2 Research Centre for Prevention, Diagnosis, and Treatment of Cancer, University of Catania, 95123 Catania, Italy; 3 Unit of Oncogenetics and Functional Oncogenomics, Centro di Riferimento Oncologico di Aviano (CRO Aviano) IRCCS, National Cancer Institute, 33081 Aviano, Italy; spiccinin@cro.it; 4 Epidemiology and Biostatistics Unit, National Cancer Institute-IRCCS ‘Fondazione G. Pascale’, 80131 Naples, Italy

**Keywords:** cutaneous melanoma, targeted therapy, drug resistance, MAPK pathway, PI3K–Akt pathway

## Abstract

The targeting of the Mitogen-Activated Protein Kinase (MAPK) signalling pathway in melanoma improves the prognosis of patients harbouring the V-Raf Murine Sarcoma Viral Oncogene Homolog B1 (BRAF) mutation. However, a fraction of these patients may experience tumour progression due to resistance to targeted therapy. Mutations affecting the Phosphoinositol-3-Kinase (PI3K)–Akt pathway may favour the onset of drug resistance, suggesting the existence of a crosstalk between the MAPK and PI3K–Akt pathways. We hypothesized that the inhibition of both pathways may be a therapeutic option in resistant melanoma. However, conflicting data have been generated in this context. In this study, three different A375 cell melanoma models either overexpressing or not expressing the wild-type or mutated form of the PhosphatidylInositol-4,5-bisphosphate 3-Kinase Catalytic Subunit Alpha (*PIK3CA*) gene were used to clarify the therapeutic response of melanoma to BRAF, Mitogen-Activated Protein Kinase Kinase 1 (MEK), and PI3K inhibitors in the presence of the PIK3CA H1047R mutation. Our data strongly support the notion that the crosstalk between the MAPK and PI3K–Akt pathways is one of the main mechanisms associated with melanoma development and progression and that the combination of MAPK and PI3K inhibitors may sensitize melanoma cells to therapy.

## 1. Introduction

Despite the evolution of anticancer pharmacological treatments with the development of novel targeted therapies and the introduction of immunotherapies, cutaneous melanoma still represents one of the most lethal tumours worldwide [1,2,3]. The high aggressiveness and metastatic power of melanoma as well as its resistance mechanisms to therapies are mediated by the overexpression of oncogenes and proteases that favour tumour invasiveness [4,5] and the overactivation of different signalling transduction pathways [6].

To effectively counteract these negative prognostic behaviours, novel therapeutic strategies are adopted. In particular, the targeting of the RAF–MEK–ERK pathway, also known as the Mitogen-Activated Protein Kinase (MAPK) pathway, with the concomitant administration of V-Raf Murine Sarcoma Viral Oncogene Homolog B1 (BRAF) and Mitogen-Activated Protein Kinase Kinase 1 (MEK) inhibitors more effectively delays the emergence of adaptive and acquired resistance mechanisms compared with treatments with either BRAF or MEK inhibitors as single agents [7]. The results achieved by using a combined treatment with BRAF and MEK inhibitors led to a significant improvement in the progression-free survival (PFS) and overall survival (OS) of patients with BRAF-mutant advanced melanoma [8,9]. However, reactivation of MAPK signalling was observed in approximately 70% of melanomas after treatment with BRAF inhibitors, MEK inhibitors, or both [10]. In addition, 4% of melanomas resistant to BRAF inhibitors showed mutations in Akt, Phosphatase and Tensin Homolog (PTEN), and Phosphoinositol-3-Kinase (PI3K), along with other regulatory genes of the PI3K–Akt pathway associated with the development of drug resistance [11]. Therefore, the targeting of both MAPK and PI3K–Akt pathways may represent an effective strategy for improving melanoma patients’ clinical response [12].

Previous studies questioned the direct role of PI3K–Akt pathway activation in the acquired resistance to anti-BRAF therapy. Melanomas with PI3K–Akt-activating mutations showed that the reactivation of the MAPK pathway is the main mechanism of resistance to BRAF inhibitors [13,14,15]. However, there is still a need to clarify the role of PI3K–Akt-activating mutations in melanoma after the inhibition of the MAPK pathway by focusing on the understanding of the interaction between the MAPK and PI3K–Akt pathways.

We evaluated the activation of the MAPK and PI3K–Akt pathways in human BRAF V600E mutant melanoma cells (A375) transduced with a PhosphatidylInositol-4,5-bisphosphate 3-Kinase Catalytic Subunit Alpha (PIK3CA) coding sequence that either harboured or did not harbour the H1047R mutation to establish if the PIK3CA mutation is directly involved in melanoma drug resistance and if it may represent a novel effective therapeutic target for resistant melanoma.

**Figure 1:**
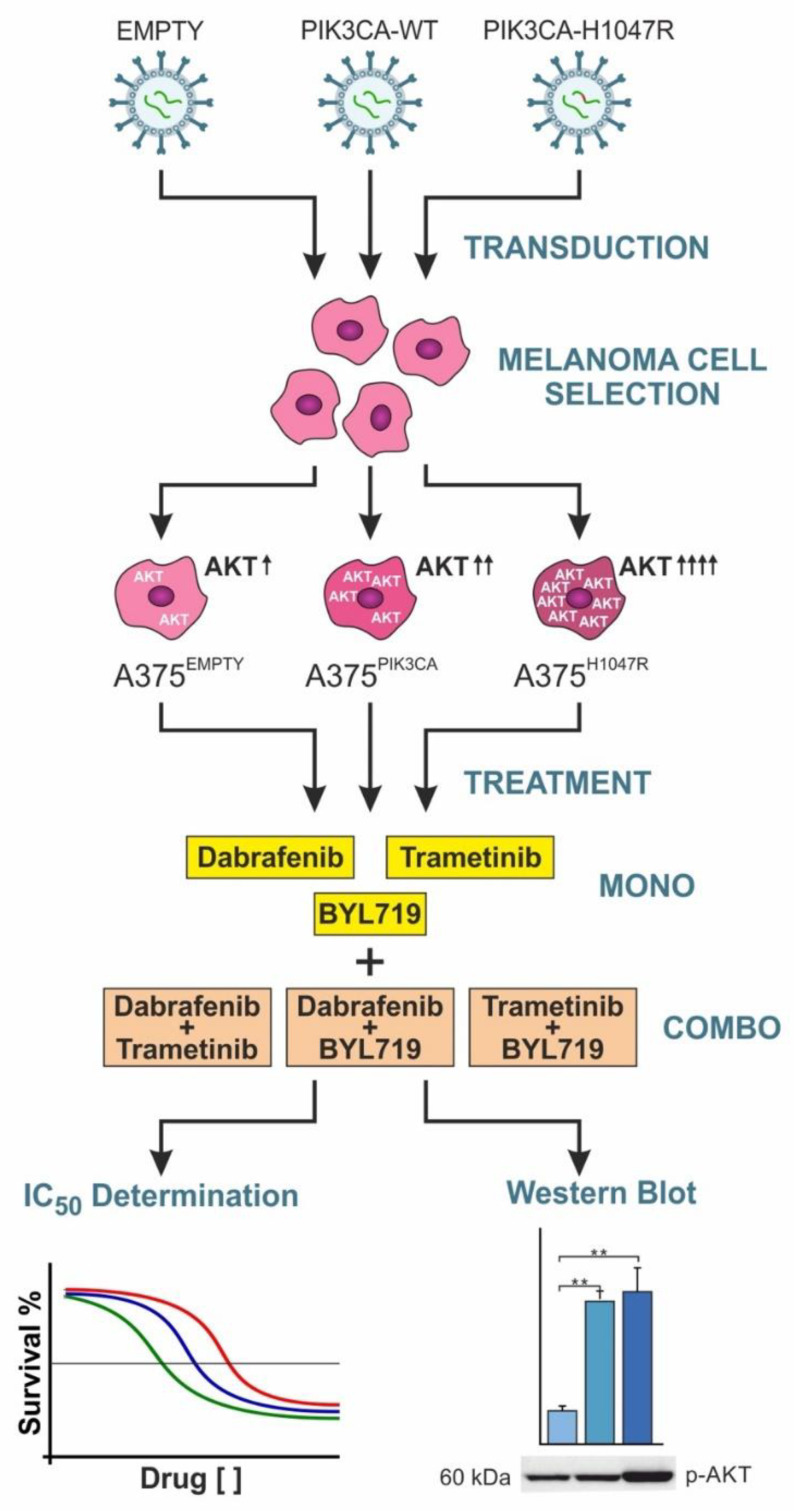
Experimental workflow of the study.

We performed functional experiments on a melanoma model to evaluate the role of the PIK3CA H1047R mutation, a main enhancer of the Akt pathway, in drug resistance and evaluate the crosstalk existing between the MAPK and PI3K–Akt pathways (Figure 1).

## 2. Materials and Methods

### 2.1. Cell Lines and Retroviral Transduction

The A375 melanoma cell line was purchased from the American Type Culture Collection (ATCC) (Rockville, MD, USA). The 293-Linx-A retroviral packaging cell line was already available at the National Cancer Institute of Aviano. RPMI-1640 and DMEM media, supplemented with L-glutamine (2 mmol/L), penicillin (100 IU), streptomycin (100 mg/mL), and 10% fetal bovine serum (FBS) (all provided from Lonza, Walkersville, MD, USA) were used for A375 and 293-Linx-A cell lines, respectively. The cells were maintained in a humidified incubator at 37 °C with an atmosphere of 5% CO_2_.

To obtain A375 cell lines harbouring the PIK3CA H1047R mutation, the retroviral vector pLNCX2-PIK3CA^H1047R^ was used. A pLNCX2-PIK3CA^wt^ vector, containing wild-type PIK3CA sequence, was used to induce PIK3CA overexpression. In addition, an empty pLNCX2 vector (pLNCX2^empty^) was used as an internal control. The pLNCX2-PIK3CA^wt^ and pLNCX2-PIK3CA^H1047R^ were obtained by cloning PIK3CA^wt^ and PIK3CA^H1047R^ inserts from pBabe puro HA PIK3CA (Plasmid #12522—Addgene) and pBabe puro HA PIK3CA H1047R (Plasmid #12524—Addgene) plasmids, respectively, into pLNCX2 plasmid.

Retroviral particles were obtained using the packaging 293-Linx-A cell line. The 293-Linx-A cells were seeded at a density of 5 × 10^5^ cells per well in a 6-well plate and grown in standard conditions. The next day, the cells were transduced with the pLNCX2-PIK3CA^H1047R^, pLNCX2-PIK3CA^wt^, and pLNCX2^empty^ retroviral vectors using Attractene transfection reagent (Qiagen, Hilden, Germany) according to the manufacturer’s protocol. The retroviral plasmid, pLNCX2-GFP, available in our lab, was used to evaluate the transfection efficiency. After 24 h, the medium was replaced, and 293-Linx-A cells were maintained at 32 °C for 24 h in a humidified incubator at 5% CO_2_. After 48 h, an equal volume of fresh complete RPMI-1640 medium supplemented with a polybrene solution (final concentration 4 μg/mL) was added to each filtered supernatant. Then, 4 mL of each lentiviral mixture was added separately to A375 cells grown overnight in a 6-well plate to reach 50% of confluency. Subsequently, spinfection was performed by centrifugation at 12,000× *g* for 60 min. The next day, transduced A375 cells were selected by using Geneticin (G-418) at a final concentration of 600 μg/mL.

### 2.2. Cell Growth and Cell Viability Assays

MTT assays were performed at 12, 24, 48, and 72 h to evaluate the growth rate of A375 pLNCX2^empty^, A375 pLNCX2-PIK3CA^wt^, and A375 pLNCX2-PIK3CA^H1047R^.

To evaluate the IC_50_ doses of dabrafenib (cat. n. S2807), trametinib (cat. n. S2673), and BYL719 (cat. N. S2814) (all purchased from Selleckchem, USA), MTT assays were performed for A375 pLNCX2^empty^, A375 pLNCX2-PIK3CA^wt^, and A375 pLNCX2-PIK3CA^H1047R^ cells. Each transduced cell line was seeded in 96-well plates at density of 3 × 10^3^ cells per well prior to treatment with serial dilutions of dabrafenib (0.01–0.1–1–10–100–1000 nM), trametinib (0.001–0.01–0.1–1–10–100 nM), and BYL719 (0.001–0.01–0.1–1–10–100 μM). DMSO was used as vehicle control. A similar approach was adopted to evaluate the cell viability of transduced cells treated with a combination of dabrafenib, trametinib, and BYL719 as described in Table 1. These combined treatments were obtained by separately adding a fixed dose of BYL719 (1 μM) and trametinib (0.5 μM) to increasing doses of dabrafenib (Table 1). Similarly, dabrafenib (1 nM) and trametinib (0.5 nM) were added to the serial dilutions of BYL719. Finally, dabrafenib (0.5 nM) and BYL719 (1 μM) were added to trametinib serial dilutions (Table 1).

### 2.3. Treatment of the Transduced A375 Cells

A375 pLNCX2^empty^, A375 pLNCX2-PIK3CA^wt^, and A375 pLNCX2-PIK3CA^H1047R^ were seeded in 60 mm cell culture dishes (Thermo Fisher Scientific, Waltham, MA, USA) at a density of 3 × 10^5^ cells. After 24 h, each infected cell line was treated with increasing concentrations of the selected drugs: dabrafenib (0.0–0.5–2.0 nM), BYL719 (0.0–0.25–1.0 μM), and trametinib (0.0–0.5–1.0 nM). Combined treatments were obtained as follows: trametinib (0.0–0.25–0.5 nM) and dabrafenib (1.0 nM) for each trametinib dilution; BYL719 (0.0–0.25–1.0 μM) and dabrafenib (1.0 nM); and BYL719 (0.0–0.25–1.0 μM) and trametinib (0.5 nM). DMSO was used as control. All cell lines were treated for 48 h in standard culture conditions.

For each treatment, adherent cells were collected by scraping. Cell pellets were collected by centrifugation and stored at −80 °C. All experiments were conducted in triplicate.

Fixed doses of dabrafenib or trametinib in combined treatments (BYL719/Dabrafenib and BYL719/Trametinib, respectively) were used to evaluate the inhibitory responsiveness of MAPK pathway under gradual modulation of Akt signalling through different doses of BYL719. Similarly, increasing doses of trametinib were adopted in Trametinib/Dabrafenib treatment to investigate the effect of downstream inhibition of MAPK signalling on the cellular response to dabrafenib.

### 2.4. Western Blot

Cell pellets were lysed in NP-40 cell lysis buffer (cat. n. FNN0021—Thermo Fisher Scientific, USA) supplemented with protease and phosphatase inhibitor cocktails (Sigma Aldrich, St. Louis, MO, USA). Protein concentration was assessed using Quick Start Bradford 1× Dye Reagent assay (cat. n. 5000205—Bio-Rad Laboratories, Hercules, CA, USA). For each sample, 30 μg of proteins was separated through electrophoresis on 4% to 15% Mini-Protean TGX precast gels (cat. n. 4561083—Bio-Rad Laboratories, USA). Bio-Rad Trans-Blot Turbo was used to transfer the gel proteins into a PVDF–nitrocellulose membrane (Bio-Rad Laboratories, USA).

Western blot analysis was carried out to evaluate the amount of PIK3CA protein expression in transduced cells using the PI3 Kinase p110α (C73F8) Rabbit mAb (#4249—Cell Signaling Technology, Danvers, MA, USA) to assess transduction efficiency.

Primary antibodies, i.e., PhosphoDetect Anti-MAP Kinase ERK1/ERK2 (pThr202/pThr204) rabbit Ab (cat. n. 442685) and Anti-MAP Kinase ERK1/ERK2 rabbit Ab (cat. n. 442704) (both purchased from Darmstadt, Germany), were used to detect phosphorylated and total ERK 1 and 2 proteins according to the manufacturer’s instructions. The phospho-Akt (Ser473) rabbit antibody (#9271) and Akt rabbit antibody (#9272) (both from Cell Signaling Technology, Danvers, MA, USA) were used to detect phosphorylated and total Akt protein levels, respectively. GAPDH and H3 housekeeping proteins were detected by using GAPDH (A-3) mouse Ab (sc-137179—Santa Cruz Biotechnology, Dallas, TX, USA) and Anti-Histone H3 rabbit Ab (ab1791—Abcam, Cambridge, UK), respectively. All primary antibodies were used at a 1:1000 dilution.

Chemiluminescent detection was performed using the Bio-Rad ChemiDoc Touch Imaging System, and protein expression was analysed with ImageJ software (National Institutes of Health, Bethesda, MD, USA). All Western blot experiments were performed in triplicate.

### 2.5. Statistical Analysis

Student’s *t*-test was performed to compare differences among each treated cell line. A value of *p* ≤ 0.05 (by a two-tailed test) was considered statistically significant. IC_50_ values and cell viability graphs were obtained using GraphPad Prism V.6.

## 3. Results

### 3.1. PIK3CA H1047R Mutation Induces PI3K–Akt Pathway Activation and A375 Proliferation

**Figure 2:**
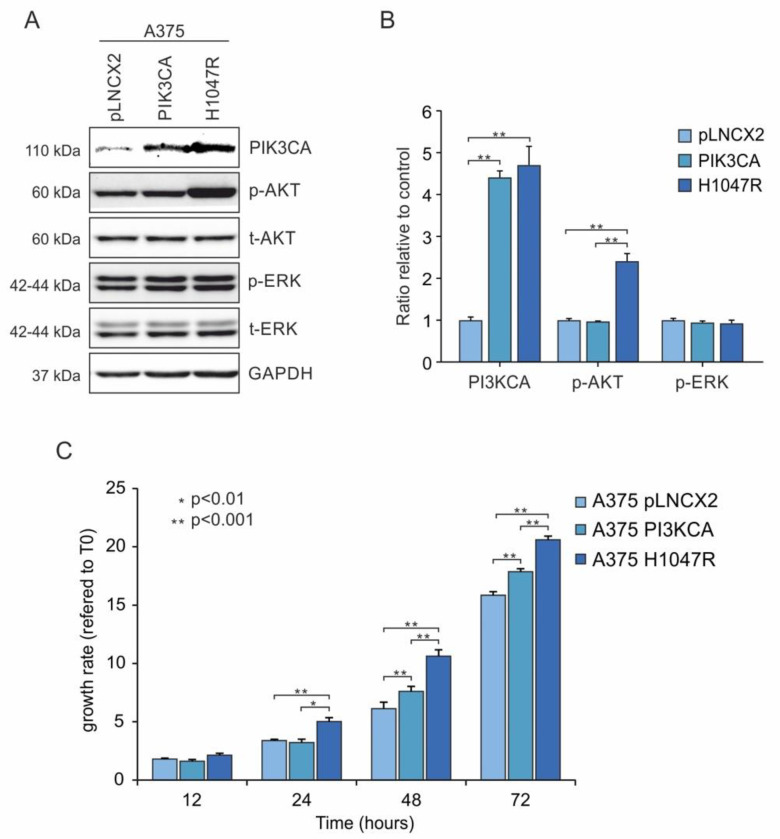
Transduction efficiency of retroviral infection and cell growth analysis. (**A**) Western blot analysis of A375^H1047R^ compared with that of A375^PIK3CA^ and A375^pLNCX2^. (**B**) Protein levels of p-ERK, p-Akt, and total ERK; Akt was evaluated to verify the transduction efficiency. (**C**) Cell growth was evaluated by MTT assay at 12, 24, 48, and 72 h. All experiments were performed in triplicate. * *p* ≤ 0.05; ** *p* ≤ 0.01.

The efficacy of cell transductions was demonstrated by Western blot analyses. PIK3CA protein levels showed a fourfold increase (*p* ≤ 0.05) in both A375^PIK3CA^ and A375^H1047R^ cells relative to the increase of an A375^pLNCX2^ control. In addition, A375^H1047R^ cells showed a higher activation (twofold increase, *p* ≤ 0.05) of p-Akt compared to A375^PIK3CA^ and A375^pLNCX2^ cells (Figure 2A,B). No significant variation was observed for p-ERK levels in the A375 transduced cells (Figure 2A,B). A significant increase in A375^H1047R^ cell growth was observed compared with the increases in both A375^PIK3CA^ and A375^pLNCX2^ cells at 24, 48, and 72 h. Furthermore, A375^PIK3CA^ cells showed a significant increase in cell proliferation compared with that seen in the A375^pLNCX2^ control at 48 and 72 h (Figure 2C).

### 3.2. PIK3CA H1047R Mutation in A375 Cells Induces Resistance to BRAF and MEK Inhibitors

**Figure 3:**
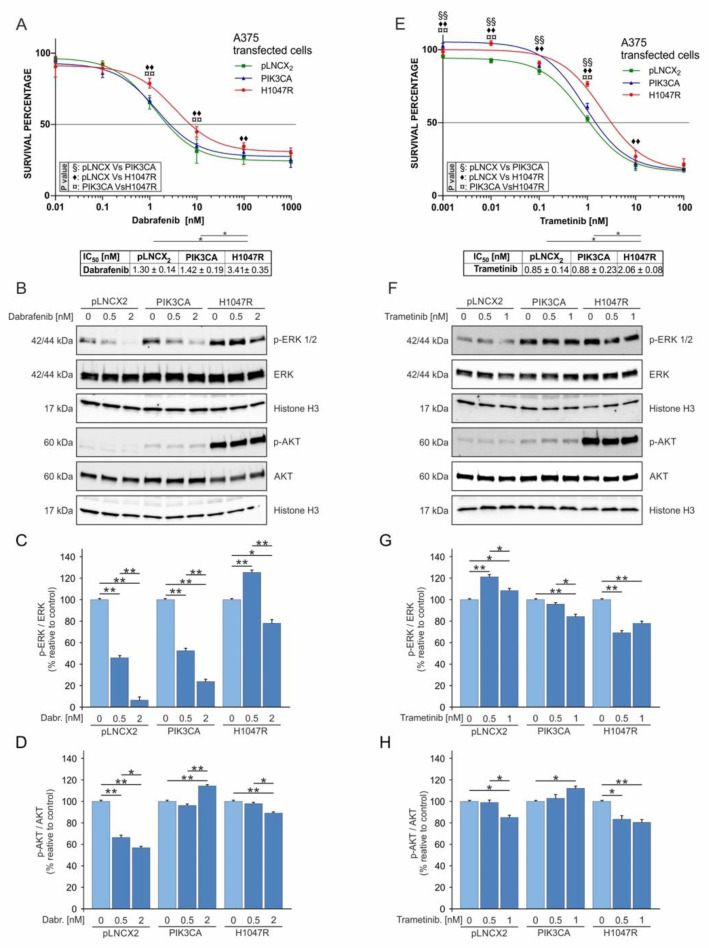
Dabrafenib and trametinib treatment of A375^pLNCX2^, A375^PIK3CA^, and A375 ^H1047R^ cells. (**A**) Cell viability evaluated through the MTT assay after 72 h of dabrafenib treatment at different concentrations (0.01–0.1–1–10–100–1000 nM). (**B**–**D**) Western blot analyses of p-ERK, p-AKT, total ERK, and total AKT protein levels and their ratio in A375 transduced cells treated with dabrafenib (0.0–0.5–2 nM) for 48 h. (**E**) Cell viability (MTT assay 72 h) of A375 clones treated with trametinib (0.001–0.01–0.1–1–10–100 nM). (**F**–**H**) Western blot analysis of p-ERK, p-AKT, total ERK, and total AKT protein levels in A375 cells treated with trametinib (0.0–0.5–1 nM) for 48 h. All experiments were performed in triplicate. * *p* ≤ 0.05; ** *p* ≤ 0.01; ◆◆, §§, ¤¤ = *p* ≤ 0.01.

An MTT analysis showed that A375^H1047R^ had a higher resistance to dabrafenib (IC_50_: 3.41 ± 0.35 nM) compared to that of A375^PIK3CA^ and A375^pLNCX2^ cells (IC_50_: 1.42 ± 0.19 nM and 1.30 ± 0.14 nM, respectively) (*p* ≤ 0.05) (Figure 3A). Similarly, A375^H1047R^ showed increased resistance to trametinib (IC_50_: 2.06 ± 0.08 nM) compared to that of other cell lines (IC_50_: 0.88 ± 0.23 nM for A375^PIK3CA^ and 0.85 ± 0.14 nM for A375^pLNCX2^) (*p* ≤ 0.05) (Figure 3E). The Western blot analysis showed a significant decrease in p-ERK levels for all A375 transduced cells in a dose-dependent manner when treated with dabrafenib. However, A375^H1047R^ cells showed only a mild reduction in p-ERK levels after treatment (Figure 3B,C). As regards p-Akt, the Western blot analysis showed a significant dose-dependent reduction in A375^pLNCX2^ cells, while weak variations were observed in the other two cell lines (Figure 3B,D).

Different results were obtained from treating all A375 transduced cells with increasing doses of trametinib. A weak reduction in both p-ERK and p-AKT levels was observed in A375^H1047R^ cells only (Figure 3F–H).

The effects of the PIK3CA H1047R mutation on MAPK and PI3K–Akt pathways were further evaluated after a treatment with two different combinations of dabrafenib and trametinib.

**Figure 4:**
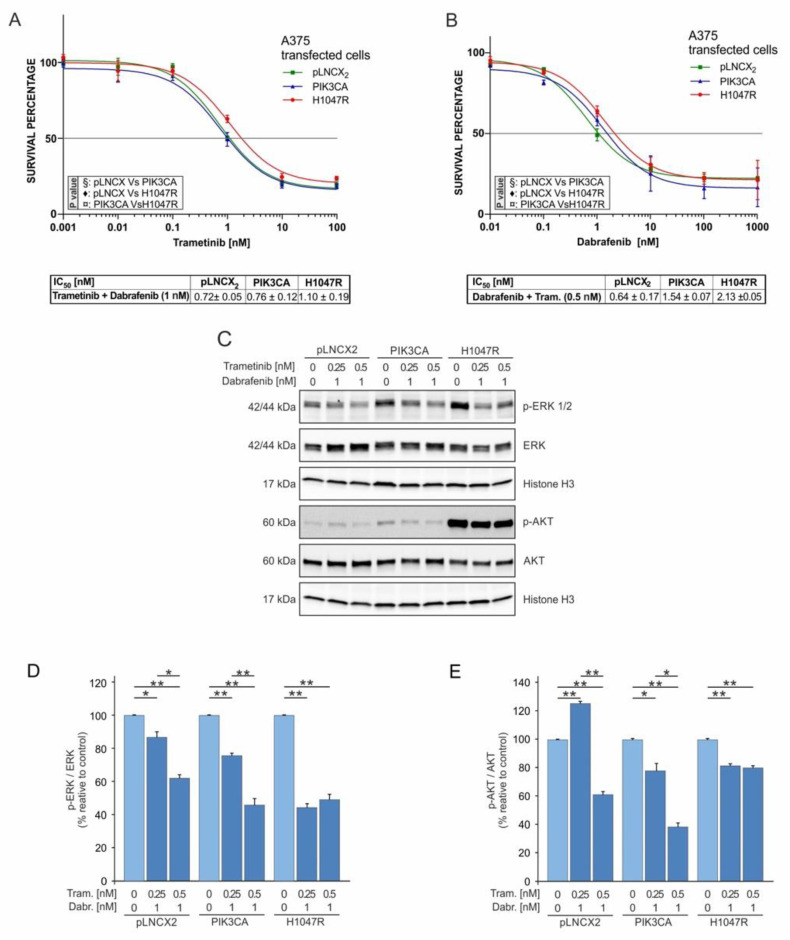
Combined dabrafenib and trametinib treatments of A375 transduced cells. (**A**) MTT assay was performed to evaluate the viability of A375 transduced cells after 72 h of treatment with 1 nM dabrafenib and increasing doses of trametinib (0.001–100 nM). (**B**) Viability (MTT assay 72 h) of A375 transduced cells treated with 0.5 nM trametinib and increasing doses of dabrafenib (0.01–100 nM). (**C**–**E**) Western blot analysis of p-ERK, p-Akt, total ERK, and total Akt protein levels in A375 transduced cells at 48 h of treatment with dabrafenib (1 nM) and increasing doses of trametinib (0.0–0.25–0.5 nM. All experiments were performed in triplicate. * *p* ≤ 0.05; ** *p* ≤ 0.01.

The survival rate of the A375^H1047R^ cell line was higher compared to that of A375^PIK3CA^ and A375^pLNCX2^ cells when treated with a fixed dose of 1 nM of dabrafenib and increasing doses of trametinib (0.001–0.01–0.1–1–10–100 nM) (Figure 4A). Similarly, by using a combined treatment with a fixed dose of trametinib (0.5 nM) and increasing doses of dabrafenib (0.01–0.1–1–10–100–1000 nM), all of the transduced cells showed similar IC_50_ values; however, the A375^H1047R^ cells were the most resistant among the transduced cells (Figure 4B). The IC_50_ values of each combined treatment (Figure 4A,B) were lower than those obtained by treating the cells with dabrafenib (Figure 3A) and trametinib (Figure 3E) alone. In particular, the A375^H1047R^ cells showed the highest reduction in IC_50_, showing a decrease of 47% for trametinib and 60% for dabrafenib (*p* ≤ 0.05).

As expected, the combined treatment with dabrafenib and trametinib induced a significant dose-dependent reduction in p-ERK in all transduced cells (Figure 4C,D). The p-ERK levels of A375^H1047R^ cells treated with both drugs were significantly reduced (p-ERK levels: 55% for Trametinib/Dabrafenib and 78% for dabrafenib; *p* ≤ 0.05) compared to those of the same cells treated with dabrafenib alone (Figure 3B,C).

Finally, p-Akt protein levels showed a consistent dose-dependent decrease only in A375^pLNCX2^ and A375^PIK3CA^ cells, while A375^H1047R^ presented a mild p-Akt reduction of approximately 20% (Figure 4C–E).

Overall, the combination of dabrafenib and trametinib in A375^H1047R^ cells resulted in the inhibition of the MAPK pathway; however, such transduced cells still showed a higher survival rate associated with p-Akt activation.

### 3.3. The Inhibition of PIK3CA H1047R Mutation Induces Dabrafenib and Trametinib Sensitivity

The treatment of the three A375 transduced cells with a PIK3CA inhibitor (BYL719) revealed different resistance patterns among cells compared with those observed when treating the cells with both dabrafenib and trametinib. In particular, the A375^H1047R^ cells showed an almost twofold greater sensitivity to BYL719 (IC_50_: 6.00 ± 0.31 µM) compared with A375^pLNCX2^ cells (IC_50_: 11.48 ± 0.43 µM), while A375^PIK3CA^ showed a moderate sensitivity to the PIK3CA inhibitor (IC_50_: 8.51 ± 0.4 µM).

**Figure 5:**
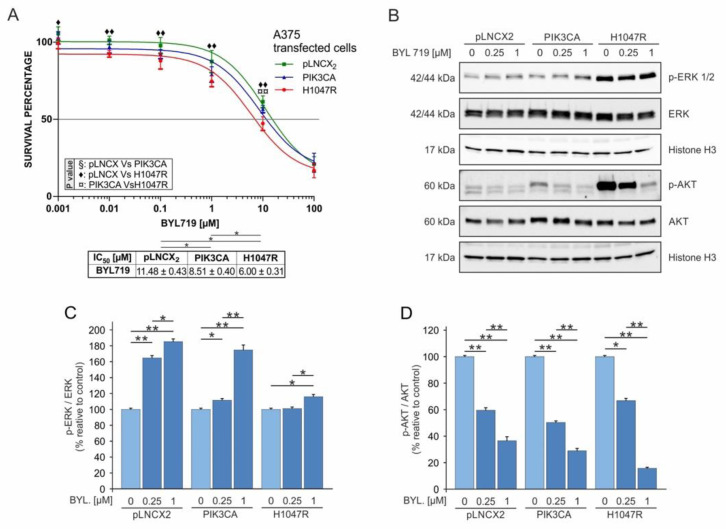
Treatment of A375^pLNCX2^, A375^PIK3CA^, and A375^H1047R^ cells with BYL719. (**A**) Cell viability evaluated by MTT assay after 72 h of treatment with BYL719 (0.001–0.01–0.1–1–10–100 μM). (**B**–**D**) Western blot analysis of p-ERK, p-Akt, total ERK, and total Akt protein levels in A375 cells treated for 48 h with BYL719 (0.0–0.25–1 μM). All experiments were performed in triplicate. * *p* ≤ 0.05; ** *p* ≤ 0.01; ◆ = *p* ≤ 0.05; ◆◆, ¤¤ = *p* ≤ 0.01.

Western blot analyses showed that BYL719 efficiently inhibited PI3K–Akt signalling in all transduced cells through the dose-dependent inhibition of p-Akt. In particular, A375^H1047R^ cells treated with 1 μM of BYL719 showed a >80% p-Akt reduction (Figure 5B,D). The A375^pLNCX2^ and A375^PIK3CA^ cells showed increased p-ERK levels after BYL719 treatment, while an opposite trend was observed for p-Akt levels (Figure 5C). Despite high residual p-Akt levels after PIK3CA inhibition of A375^H1047R^ cells, no significant difference in p-ERK levels was detected (Figure 5B–D).

To evaluate the effects of PIK3CA inhibition on dabrafenib and trametinib drug resistance in A375 cells harbouring the H1047R mutation, combined treatments using BYL719 and dabrafenib or BYL719 and trametinib were performed.

**Figure 6:**
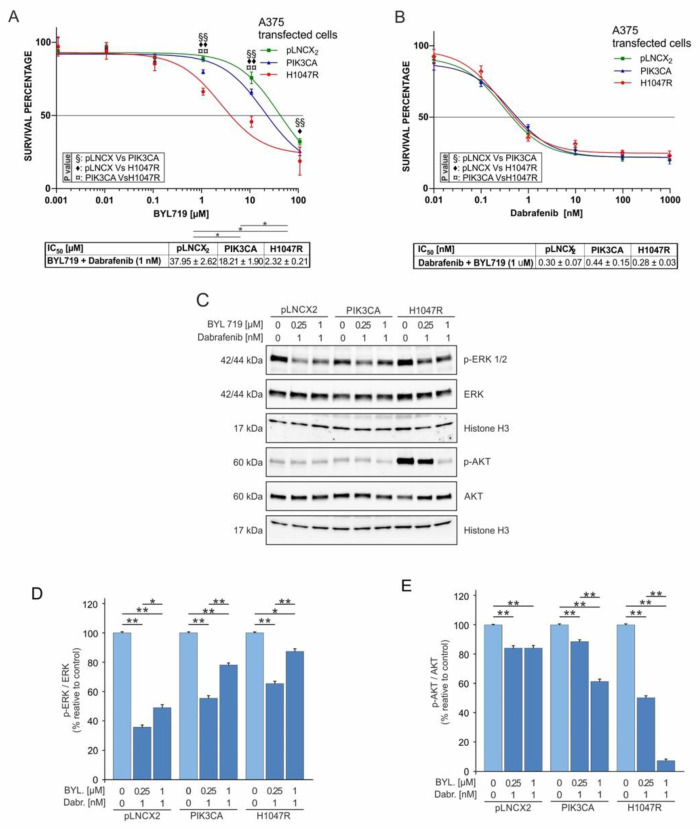
BYL719 and dabrafenib combined treatments of A375 transduced cells. (**A**) MTT assay was performed to evaluate A375 transduced cells’ viability after 72 h of treatment with 1 nM dabrafenib and increasing doses of BYL719 (0.001 to 100 μM). (**B**) Cell viability (MTT assay 72 h) of A375 transduced cells treated with 1 µM BYL719 and increasing doses of dabrafenib (0.01–1000 nM). (**C**–**E**) Western blot analyses of p-ERK, p-Akt, total ERK, and total Akt protein levels evaluated in A375 transduced cells treated for 48 h with 1 nM dabrafenib and growing doses of BYL719 (0.0–0.25–1 μM). All experiments were performed in triplicate. The statistical significance of the two-tailed Student’s *t*-test was in reference to the control. * *p* < 0.05; ** *p* < 0.01; ◆ = *p* ≤ 0.05; ◆◆, §§, ¤¤ = *p* ≤ 0.01.

The MTT assay showed that the cell viability of A375^H1047R^ was strongly reduced compared with that of A375^PIK3CA^ and A375^pLNCX2^ cells (IC_50_: 2.32 ± 0.21 µM vs. 18.21 ± 1.90 µM and 37.95 ± 2.62 µM, respectively) at BYL719 concentrations higher than 0.1 µM and a fixed concentration of dabrafenib of 1 nM, demonstrating the highest sensitivity of A375^H1047R^ to BYL719 (Figure 6A).

Interesting results were achieved by fixing the BYL719 dose at 1 µM (low dose) and varying the concentration of dabrafenib (from 0.01 to 1000 nM).

As shown in Figure 5B, the cell viability of all transduced cells was globally reduced as dabrafenib IC_50_ values ranged from 0.28 to 0.44 nM (Figure 5B) compared with those observed when cells were treated with dabrafenib alone (range: 1.30–3.41 nM) (Figure 2A). In addition, the combined therapy with BYL719 and dabrafenib reduced the drug resistance observed for A375^H1047R^, which showed an IC_50_ value similar to that of the other two cell lines (Figure 6B).

As demonstrated by Western blot analyses, BYL719 induced a reduction in p-Akt protein levels in a dose-dependent manner; such a reduction was more evident for A375^H1047R^ cells, which had higher baseline p-Akt levels (Figure 6C,E) and were the most sensitive to BYL719. The protein levels of p-ERK showed a low reduction at high BYL719 concentrations to balance the p-Akt reduction. In particular, the A375^PIK3CA^ and A375^H1047R^ cells showed lower p-ERK reduction compared with that seen in A375^pLNCX2^ as a consequence of high p-Akt levels (Figure 6C–E).

**Figure 7:**
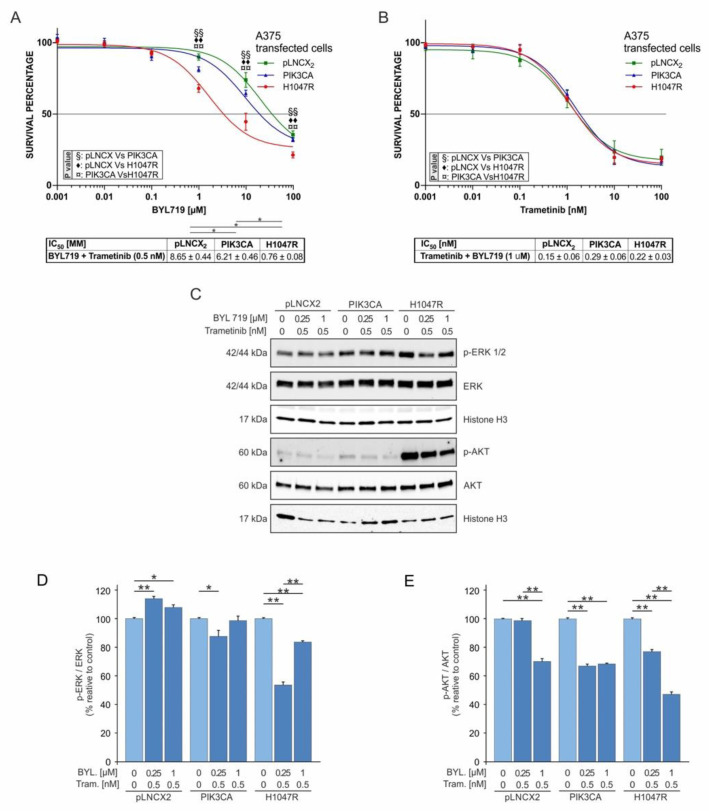
BYL719 and trametinib combined treatment of A375 transduced cells. (**A**) Cell viability (MTT assay 72 h) of A375 transduced cells treated with 0.5 nM trametinib and increasing doses of BYL719 (0.001–100 μM) for 72 h. (**B**) MTT assay was performed to evaluate A375 transduced cells’ viability after treatment with 1 µM BYL719 and increasing doses of trametinib (0.001–100 nM) for 72 h. (**C**–**E**) p-ERK, p-Akt, total ERK, and total Akt protein levels in A375 transduced cells treated with 0.5 nM trametinib and growing doses of BYL719 (0.0–0.25–1 μM) at 48 h were evaluated by Western blot. All experiments were performed in triplicate. The statistical significance of the two-tailed Student’s *t*-test was in reference to the control. * *p* < 0.05; ** *p* < 0.01; ◆◆, §§, ¤¤ = *p* ≤ 0.01.

Moreover, the combination of increasing doses of BYL719 and a fixed dose of trametinib (0.5 nM) showed a consistent reduction in A375^H1047R^ viability (Figure 7A), which showed a similar trend to that observed by combining a fixed dose of dabrafenib with BYL719 (Figure 6A). Conversely to what was observed for the Dabrafenib/BYL719 combined treatment, p-ERK levels did not decrease in the A375^pLNCX2^ and A375^PIK3CA^ cells but only in the A375^H1047R^ cells (Figure 7C,D). As regards p-Akt protein levels, the Trametinib/BYL719 combined treatment significantly reduced p-Akt in all cell lines to a lesser extent compared to the Dabrafenib/BYL719 treatment (Figure 7C,E).

Finally, as shown in Figure 6B, the combined treatment with increasing doses of trametinib and a fixed dose of BYL719 (1 µM) reverted the drug resistance to therapy observed for A375^PIK3CA^ and A375^H1047R^ as no difference was observed between the IC_50_ values calculated for each cell line (Figure 7E). Furthermore, these IC_50_ values were significantly lower (Trametinib/BYL719 range: 0.29–0.15 nM; trametinib range: 2.06–0.85 nM; *p* ≤ 0.05) than those observed when treating the cells with trametinib alone (Figure 3E).

Supporting our initial hypothesis of a crosstalk existing between the MAPK and PI3K–Akt pathways, the data reported in Figure 6D and Figure 7D demonstrate that the inhibition of the PI3K–Akt pathway operated by BYL719 led to the reactivation of the MAPK pathway, even in the presence of dabrafenib or trametinib. In particular, the reduction in the pERK/ERK ratio was lower at 1 µM of BYL719 and higher at 0.25 µM of BYL719 in both combined treatments with Dabrafenib/BYL719 and Trametinib/BYL719.

## 4. Discussion

**Figure 8:**
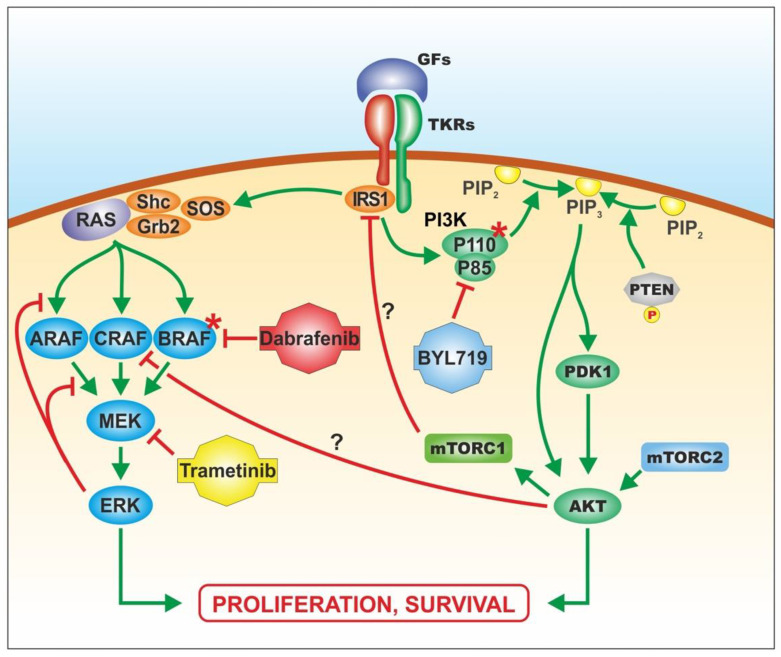
Crosstalk between the MAPK and PI3K–Akt pathways and interaction between BRAF-, MEK-, and PI3K-selective inhibitors with their molecular targets. * = Activating mutation.

Combined treatments with BRAF and MEK inhibitors have improved the prognosis of melanoma patients compared to monotherapy with BRAF inhibitors alone [8,16,17,18]. However, clinical studies have demonstrated that a significant fraction of refractory patients experience tumour progression six months after the initiation of such treatments [9,16]. Although BRAF V600E is a dominant driver of mutations that are widely associated with melanoma aggressiveness [19], a small portion of resistant melanomas take advantage of the activation of the compensatory PI3K–Akt signalling cascade sustained by different genetic alterations. PI3K–Akt pathway activation due to genetic or epigenetic events is associated with drug resistance in different tumours [20,21]. As regards melanoma, activation of the PI3K–Akt pathway promotes resistance mechanisms to both BRAF and MEK inhibitors [22]. This statement is further supported by findings demonstrating a crosstalk existing between the overactivated PI3K–Akt pathway and MAPK pathway in resistant melanoma responsible for the fine regulation of these pathways, including cross-inhibition mechanisms [15,23], that may be central for cellular responses to proliferative and survival stimuli [24] (Figure 8).

As the MAPK and PI3K–Akt pathways may positively or negatively regulate mutual activities, the combination of BRAF or MEK inhibitors with a PI3K–Akt inhibitor was proposed to overcome the resistance to MAPK inhibitors in BRAF V600E mutant tumours [7]. However, the mechanisms by which such combinations may revert resistance to targeted therapy are still debated.

Accordingly, the detection of PI3K–Akt mutations in melanomas may occur in BRAF (V600E/K)-mutated tumours conferring resistance to therapies [10]. Although some studies demonstrated that PI3K–Akt pathway activation is associated with BRAF inhibitor resistance in preclinical models, PI3K–Akt pathway alterations do not necessarily affect the clinical response [25,26]. For example, it was observed that several resistant tumours harbouring PIK3CA mutations affected the therapeutic efficacy of anti-MAPK drugs. Of these, one of the most represented PIK3CA mutations (affecting 4% of melanoma patients, according to COSMIC data) with a pathogenetic role in drug resistance is the PIK3CA H1047R mutation associated with both anti-BRAF and anti-MEK inhibitors of drug resistance [27,28,29].

We hypothesized that the concomitant targeting of both MAPK and PI3K–Akt pathways in a PIK3CA-mutated melanoma model resistant to BRAF and MEK inhibitors would result in a better therapeutic efficacy that reverts drug resistance mechanisms. To the best of our knowledge, there are no studies on BRAF-mutated melanoma cells where the PIK3CA mutation was artificially induced and concomitantly treated with BRAF/PI3K or MEK/PI3K inhibitors. We analysed the behaviour of the MAPK and PI3K–Akt pathways in the presence of the PIK3CA H1047R mutation in melanoma cells treated with targeted therapy. The overexpression of both PIK3CA wild-type and PIK3CA H1047R was induced in A375 melanoma cells. The transduced cells (A375^pLNCX2^, A375^PIK3CA^, and A375^H1047R^) were treated with BRAF and MEK inhibitors in combination or not in combination with the PI3K inhibitor BYL719 to assess if the PIK3CA H1047R mutation is associated with drug resistance and if its targeting reverses resistance to approved targeted therapies. Cell viability assays demonstrated that the transduced A375 melanoma cells with an active PI3K–Akt pathway (A375^PIK3CA^ and A375^H1047R^) showed a higher proliferation rate compared to that of A375^pLNCX2^, suggesting that the activation of the PI3K–Akt pathway is involved in melanoma progression. In addition, by treating these transduced cells with both BRAF and MEK inhibitors, we demonstrated that activation of the PI3K–Akt pathway mediated by the PIK3CA H1047R mutation confers drug resistance to dabrafenib and trametinib. In particular, the activation of the PI3K–Akt pathway reduced the inhibitory effects of dabrafenib toward p-ERK, conferring a survival advantage to the cells. These data suggested that the existence of a crosstalk between the MAPK and PI3K–Akt pathways was responsible for the minor reduction of p-ERK levels and the increase in resistance to anti-BRAF treatment observed for A375^H1047R^ cells.

BRAF and MEK inhibitors are currently used as a combined targeted therapy for melanoma patients to reduce possible therapeutic failure due to the activation of other pathways, including PIK3–Akt, or to the onset of other mutations occurring in the MAPK pathway [30,31]. In our study, dabrafenib and trametinib were used as inhibitors for BRAF and MEK, respectively. These drugs were selected as they are most widely used in clinical practice [32]. Similarly, BYL719 was selected as the PI3K inhibitor as it is widely used for the treatment of PI3K-mutated tumours [33].

To further establish the impact of the PIK3CA H1047R mutation on the crosstalk between the MAPK and PI3K–Akt pathways as well as on the drug resistance observed in patients treated with both dabrafenib and trametinib, combined treatments using these two drugs were performed in the transduced cells. The results revealed that both combined treatments significantly reduced the IC_50_ doses of all cells that were more sensitive to the treatments. Although all transduced cells were more sensitive to the combined treatments, the A375^H1047R^ cells exhibited higher resistance compared to that of the other cell lines. Conversely to dabrafenib treatment, the combined Dabrafenib/Trametinib treatment induced a greater reduction in p-ERK and p-Akt in A375^H1047R^ cells, suggesting that the reduced resistance of these cells (IC_50_: 3.41 ± 0.35 nM Dabr. vs. 2.13 ± 0.05 nM Dabr. + 0.5 nM Tram; IC_50_: 2.06 ± 0.08 nM Tram. vs. 1.10 ± 0.19 nM Tram. + 1 nM Dabr.) was related to the inactivation of both MAPK and PI3K–Akt pathways. In the combined treatments, the highest survival rate of A375^H1047R^ cells may have been related to the highest p-Akt levels, supporting the notion that the overactivation of the PI3K–Akt pathway balances the inhibition of the MAPK pathway mediated by both dabrafenib and trametinib [15,22].

The importance of PI3K–Akt pathway activation associated with resistance to MAPK inhibitors was further demonstrated through the direct inhibition of this pathway mediated by the treatment with BYL719. p-Akt levels strongly decreased in a dose-dependent manner after treatment with BYL719. In addition, such a decrease was more evident in A375 transduced cells that had overactivation of the PI3K–Akt pathway (A375^PIK3CA^ and A375^H1047R^). As a consequence of p-Akt inhibition, drug resistance to MAPK inhibitors was reverted. When treated with BYL719 alone, all transduced cells showed increased levels of p-ERK, suggesting that the MAPK and PI3K–Akt pathways interact with each other and, in particular, when p-Akt is inhibited, p-ERK increases to compensate for the inactivation of the PI3K–Akt pathway.

To elucidate the functional effects of the crosstalk between the MAPK and PI3K–Akt pathways and the therapeutic potential of the concomitant inhibition of these two pathways both in the presence and absence of the PIK3CA H1047R mutation, combined treatments using dabrafenib and BYL719 or trametinib and BYL719 were performed. Our results confirmed the therapeutic potential of the concomitant inhibition of the MAPK and PI3K–Akt pathways. The A375^H1047R^ cells presented an IC_50_ value similar to or lower than that observed for A375^pLNCX2^ and A375^PIK3CA^ cells when treated with a fixed dose of BYL719 and increasing doses of dabrafenib or trametinib or vice versa, suggesting that the concomitant inhibition of the two pathways effectively counteracts drug resistance due to acquired mutations affecting the PI3K–Akt pathway. In addition, for all transduced cells, the IC_50_ doses of dabrafenib showed a 3.3- to 12.1-fold reduction when a fixed dose of BYL719 was simultaneously administered, and the greatest reduction was observed for the A375^H1047R^ cells (IC_50_: 3.41 ± 0.35 nM vs. 0.28 ± 0.03 nM) that were previously resistant to treatment. Similarly, the IC_50_ dose of trametinib showed an average sixfold reduction in the three transduced cells treated with increasing doses of trametinib and a fixed dose of BYL719. These drug combinations allowed for the reversal of the drug resistance observed for A375^H1047R^ as the IC_50_’s of all three transduced cells were similar. In addition, a significant IC_50_ reduction in BYL719 was observed only for the A375^H1047R^ cells when using increasing doses of BYL719 and a fixed dose of dabrafenib or trametinib.

Finally, the concomitant targeting of BRAF and PI3K or MEK and PI3K further demonstrated the existing crosstalk between the MAPK and PI3K–Akt pathways. The protein expressions of p-ERK and p-Akt in all treated transduced cells had opposite trends. When p-Akt was significantly downregulated by increasing doses of BYL719, p-ERK levels increased or remained stable as a compensatory mechanism of PI3K–Akt inhibition, despite the presence of dabrafenib or trametinib. Similarly, as increasing doses of dabrafenib led to a reduction of p-ERK, p-Akt levels increased in a dose-dependent manner; however, the presence of a fixed dose of BYL719 led to low levels of p-Akt.

## 5. Conclusions

Our results strongly suggest that there exist compensatory mechanisms between the MAPK and PI3K–Akt pathways when they are selectively inhibited using specific targeted inhibitors. Our functional experiments highlighted that concomitant inhibition of the two pathways is fundamental in the presence of acquired resistance due to PI3K–Akt-activating mutations.

Taken together, our data showed that PI3K–Akt activation mediated by the PIK3CA H1047R mutation promotes the proliferation and resistance of melanoma cells treated with BRAF and MEK inhibitors. In addition, MAPK and PI3K–Akt pathways interact with each other to compensate for the inhibition of p-ERK or p-Akt mediated by dabrafenib or trametinib and BYL719, respectively. This compensatory mechanism results in an increase in p-ERK when p-Akt is inhibited and vice versa.

The identification of resistance mechanisms associated with the PIK3CA H1047R mutation and the definition of crosstalk existing between the MAPK and PI3K–Akt pathways demonstrated in this study further encourage the adoption of combined treatments with both MAPK- and PI3K–Akt-selective inhibitors that are useful for sensitizing melanoma cells to therapy.

Our results represent proof of a concept that helps us understand the crosstalk existing between the MAPK and PIK3CA pathways that is potentially involved in melanoma drug resistance. Proteomic analyses and bioinformatics approaches, including machine learning processes, may be useful to further validate our results through the identification of other key signalling proteins involved in the cellular drug response. Therefore, further experiments should be performed using protein array approaches (e.g., RPPA array) and high-throughput molecular analyses to fully characterize the crosstalk existing between the two pathways as well as any protein–protein interactions with other molecular pathways involved in both melanoma progression and drug resistance mechanisms.

## Figures and Tables

**Table 1:** Cell lines treatments.

Drugs	Dabrafenib	Trametinib	BYL719
Dabrafenib (0.01–0.1–1–10–100–1000 nM)	/	0.5 nM	1 μM
Trametinib (0.001–0.01–0.1–1–10–100 nM)	0.5 nM	/	1 μM
BYL719 (0.001–0.01–0.1–1–10–100 μM)	1 nM	0.5 nM	/

## Data Availability

The data generated in this study were deposited in the Zenodo platform (https://doi.org/10.5281/zenodo.5814964) (created and accessed on 31 October 2021) and are also available from the corresponding author upon reasonable request.
